# Supplementary material for: Association of Helicobacter pylori as an Extragastric Reservoir in the Oral Cavity with Oral Diseases in Patients with and Without Gastritis—A Systematic Review
Source: Microorganisms. 2025 Aug 21;13(8):1955. doi: 10.3390/microorganisms13081955 (PMC12388271; doi:10.3390/microorganisms13081955)
Supplement: Supplementary file 1 [file microorganisms-13-01955-s001.zip › microorganisms-3776844-supplementary.pdf]

microorganisms

MDPI

Supplementary Materials

Table S1. Complementary materials. Preferred Reporting Item Checklist for Systematic Reviews and Meta-Analyses (PRISMA) 2020 guidelines.

Section and Topic	Arti-cle #	Checklist Item	Location where the article is reported
**TITLE**			
Title	1	Identify the report as a systematic review.	p. 1
**ABSTRACT**			
Abstract	2	Check out the PRISMA 2020 summary checklist.	p. 1
**INTRODUCTION**			
Foundation	3	Describe the rationale for the review in the context of existing knowledge.	pp. 1-3
Objectives	4	Provide an explicit statement of the objectives or questions that the review addresses.	p. 4
**METHODS**			
Eligibility Criteria	5	Specify the inclusion and exclusion criteria for the review and how studies were pooled for synthesis.	p. 4
Sources of infor-mation	6	Specify all databases, registries, websites, organizations, reference lists, and other sources searched or queried to identify studies. Specify the date each source was searched or last queried.	p. 4
Search strategy	7	Present complete search strategies for all databases, records, and websites, including the filters and limits used.	p. 4
Selection process	8	Specify the methods used to decide whether a study met the review's inclusion criteria, including how many reviewers examined each record and each retrieved report, whether they worked independently, and, if applicable, details of the automation tools used in the process.	p. 5

https://doi.org/10.3390/xxxxx

Section and Topic	Article #	Checklist Item	Location where the article is reported
Data collection process	9	Specify the methods used to collect data from the reports, including how many reviewers collected data from each report, whether they worked independently, any processes for obtaining or confirming data from study investigators, and, if applicable, details of the automation tools used in the process	p. 6
Data Elements	10a	List and define all the results for which data was searched. Specify whether all outcomes that were compatible with each outcome domain in each study were searched (e.g., for all measures, time points, analyses) and, if not, the methods used to decide which results to collect.	pp. 5
	10b	List and define all other variables for which data were sought (e.g. participant and intervention characteristics, funding sources). Describe any assumptions made about any missing or unclear information.	p. 5
Assessment of study risk of bias	11	Specify the methods used to assess risk of bias in the included studies, including details of the tools used, how many review authors assessed each study and whether they operated independently and, if applicable, details of the automation tools used in the process.	p. 5
Measures of effect	12	Specify for each outcome the effect(s) measure(s) (e.g., risk ratio, mean difference) used in the synthesis or presentation of the results.	p. 5
Synthesis methods	13th	Describe the processes used to decide which studies were eligible for each synthesis (e.g., tabulate the characteristics of the study intervention and compare them to the groups planned for each synthesis (item #5)).	p. 6
	13b	Describe the methods required to prepare data for presentation or synthesis, such as handling missing summary statistics or data conversions.	N/A
	13c	Describe any methods used to tabulate or visually display the results of individual studies and syntheses.	p. 5
	13d	Describe the methods used to synthesize the results and provide a rationale for the options. If a meta-analysis was performed, describe the models, methods for identifying the presence and extent of statistical heterogeneity, and the software packages used.	N/A
	13e	Describe any methods used to explore possible causes of heterogeneity among study results (e.g. subgroup analysis, meta-regression).	N/A
	13f	Describe the sensitivity analyses performed to assess the robustness of the synthesized results.	p. 5
Assessing Reporting Bias	14	Describe any methods used to assess risk of bias due to lack of results in a synthesis (arising from reporting biases).	p. 6
Certainty assessment	15	Describe any method used to assess the certainty (or confidence) in the body of evidence of a result.	N/A

Section and Topic	Article #	Checklist Item	Location where the article is reported
**RESULTS**			
Study selection	16a	Describe the results of the search and selection process, from the number of records identified in the search to the number of studies included in the review, ideally using a flowchart.	p.7
	16b	Cite studies that appear to meet the inclusion criteria, but were excluded, and explain why they were excluded.	p. 8
Study characteristics	17	Cite each included study and present its characteristics.	pp. 9 – 11
Risk of bias in studies	18	Present risk of bias assessments for each included study.	p. 8
Results of individual studies	19	For all outcomes, submit, for each study: (a) summary statistics for each group (where applicable) and (b) an estimate of the effect and its accuracy (e.g., confidence/credibility interval), ideally using structured tables or graphs.	N/A
Results of the syntheses	20a	For each synthesis, briefly summarize the characteristics and risk of bias among the studies that contributed.	p. 9
	20b	Present the results of all the statistical syntheses made. If a meta-analysis was performed, present for each meta-analysis the summary estimate and its accuracy (e.g. confidence/credibility interval) and measures of statistical heterogeneity. If you are comparing groups, describe the direction of the effect.	p. 10
	20c	Present the results of all research on possible causes of heterogeneity among study results.	p. 12
	20d	Present the results of all sensitivity analyses performed to assess the robustness of the synthesized results.	p. 12
Information biases	21	Present assessments of risk of bias due to lack of results (derived from reporting biases) for each synthesis assessed.	pp. 7-8
Certainty of the evidence	22	Present assessments of certainty (or confidence) in the body of evidence for each outcome assessed.	N/A
**DISCUSSION**			
Discussion	23a	Provide a general interpretation of the results in the context of other evidence.	pp. 12 -14
	23b	Discuss any limitations of the evidence included in the review.	pp. 12-14

Section and Topic	Article #	Checklist Item	Location where the article is reported
	23c	Discuss the limitations of the review processes used.	p. 14
	23d	Discuss the implications of the results for practice, policy, and future research.	p. 14
**OTHER INFORMATION-**			
Registration and protocol	24th	Provide registration information for the review, including the name of the review and the registration number, or indicate that the review was not registered.	p. 3
	24b	Indicate where the review protocol can be accessed or indicate that a protocol was not prepared.	p. 3
	24c	Describe and explain any changes to the information provided in the registry or protocol.	p. 3
Support	25	Describe the sources of financial or non-financial support for the review, and the role of funders or sponsors in the review.	N/A
Conflicting interests	26	Declare any competing interests of the review authors.	N/A
Availability of data, code, and other materials	27	Please report which of the following are publicly available and where they can be found: template data collection forms; data extracted from the included studies; data used for all analyses; analytical code; any other materials used in the review.	N/A
